# Supplementary material for: Eminuscent phase in frustrated magnets: a challenge to quantum spin liquids
Source: Nat Commun. 2022 May 30;13:2993. doi: 10.1038/s41467-022-30739-0 (PMC9151641; doi:10.1038/s41467-022-30739-0)
Supplement: Supplementary file 1 — Supplementary Information [file 41467_2022_30739_MOESM1_ESM.pdf]

Supplemental Material for "How frustrated magnets hide a phase transition and challenge quantum spin liquids"

Sergey Syzranov[1] and Arthur Ramirez[1]

[1]*Physics Department, University of California, Santa Cruz, California 95064, USA*

I. DETAILS OF PUBLISHED DATA PRESENTATION

The data presented in Figs. 1 and 2 were obtained by digitising data from published work, referenced in the text. Any error introduced in digitising is less than the size of the symbols in our plots. Converting susceptibility units from the conventional emu/mole to dimensionless units requires knowledge of the mass density. For the end-members of the series, we used either published density values or obtained values from the Materials Project (https://materialsproject.org/). For compounds that are solid-solutions of two end-members, we assumed a Vegard's law (linear) interpolation of the lattice constant and thus density. Values of T_g were taken from the position of the peak in zero-field-cooled susceptibility.

In Fig. 2 are plotted T_g vs. disorder, x, for those materials where disorder can be related to a chemical constituent. For most of the compound series, x represents the concentration of spin vacancies due to the introduction of non-magnetic impurity atoms[1–4], but it can also indicate substitution on a non-magnetic site[5–7]. In these cases, if the size of the impurity atoms differs significantly from that of the host atoms, as exemplified by La^{3+} in $Y_2Mo_2O_7$, chemical substitution can lead to large values of random strain, leading to a large modulation of the exchange interactions. Thus, the series $Y_{2(1-x)}La_{2x}Mo_2O_7$ is not included in our Fig. 2a survey because θ_W changes sign from antiferromagnetic to ferromagnetic as x increases from 0 to 0.3, indicating that introduction of the La^{3+} ion for Y^{3+} significantly alters the character of the exchange interactions that govern the behaviour of the background spins[8]. The compound $Zn_{1-x}Cd_xCrO_4$, where Cd^{2+} replaces Zn^{2+}, is not included in Fig. 2a due to the absence of vacancy variation. At the same time, it is included in Fig. 1, as the disorder resulting from the replacement has no effect on frustration[5,9].

As alluded to in the main text, some of the substitution series shown in Fig. 2a do not approach the pure limit. For the $(Mn,Cd)Te$ and $Zn(Cr,Ga)_2O_4$ series, the SG phase is replaced by an antiferromagnetic phase, accompanied by a change in the characteristic $1/\chi(T)$ behaviour. For the $(Fe,Ga)_2TiO_5$ series, the Fe_2TiO_5 end member has the highest possible Fe content for the

pseudobrookite structure. Randomness arises from mixing of the Fe^{3+} and Ti^{4+} ions among their respective crystallographic sites. Thus, due to the different oxidation states of Fe and Ti, increasing Fe^{3+} content beyond 2/3 is not possible within the same structure without destroying the insulating phase.

II. GLASS TRANSITION IN A WEAKLY DISORDERED SYSTEM

In this section, we estimate the glass transition temperature in a system of spins with exchange interactions with randomly located generic defects and demonstrate its vanishing in the limit of the vanishing density n_{imp} of the defects. Such defects may be represented, for example, by vacancies in the lattice of the system, spinless foreign atoms or perturbations in the exchange interaction between the spins. In order to analyse the glass transition, we employ the replica trick. The replicated Hamiltonian of the system is given by

$$\mathcal{H} = \mathcal{H}_0 + \mathcal{H}_{\text{dis}}, \tag{1a}$$

$$\mathcal{H}_0 = \frac{1}{2} \sum_{\mathbf{r},\mathbf{r}',\alpha} J_{\mathbf{r},\mathbf{r}'} s^\alpha_{\mathbf{r}} s^\alpha_{\mathbf{r}'}, \tag{1b}$$

$$\mathcal{H}_{dis} = \frac{1}{2} \sum_{i,\alpha} s^\alpha_{\mathbf{r}} K_{\mathbf{r}-\boldsymbol{\rho}_i,\mathbf{r}'-\boldsymbol{\rho}_i} s^\alpha_{\mathbf{r}'}, \tag{1c}$$

where we consider, for simplicity, classical Ising spins $s_{\mathbf{r}} = \pm 1$ labelled by their locations $\mathbf{r}$ and $\mathbf{r}'$ in the lattice and the replica index α; the Hamiltonian $\mathcal{H}_0$ describes the pure systems; $\mathcal{H}_{dis}$ accounts for the effect of the defects; $J_{\mathbf{r}\mathbf{r}'}$ is the coupling between spins at locations $\mathbf{r}$ and $\mathbf{r}'$ and is a function of only the relative position $\mathbf{r} - \mathbf{r}'$. Quenched disorder is represented by the randomness of the locations $\boldsymbol{\rho}_i$ of the defects in Eq. (1c); the matrix $K_{\mathbf{r}_1,\mathbf{r}_2}$ is non-random and describes the modifications of the couplings $J_{\mathbf{r},\mathbf{r}'}$ caused by one defect located at $\boldsymbol{\rho} = 0$.

The defects thus create identical perturbations near different locations $\boldsymbol{\rho}_i$. For example, vacancies in a lattice with nearest-neighbour exchange coupling $J_{\mathbf{r},\mathbf{r}'} = J$ are described by the elements $K_{\mathbf{r}-\boldsymbol{\rho},\mathbf{0}} = K_{\mathbf{0},\mathbf{r}-\boldsymbol{\rho}} = -J$ at all sites $\mathbf{r}$ adjacent to the location $\boldsymbol{\rho}$ of the vacancy, effectively cancelling the interactions between the spin at the location of the vacancy and the neighbouring spins, and to vanishing elements of $K_{\mathbf{r}_1,\mathbf{r}_2}$ for all other positions. If the defect is represented by a foreign atom with the same spin as the atoms in the compound, the elements of the matrix $K_{\mathbf{r}_1,\mathbf{r}_2}$ may represent small perturbations of the exchange couplings around a defect. If quenched disorder is represented by random-bond disorder on all sites coming from, e.g., random strain fields, it can be described by the same model with the defect density set to the density of the sites, $n_{\text{imp}} \sim n$.

We perform averaging of the replicated partition function of the system over the locations $\boldsymbol{\rho}_i$ of all defects, $\langle Z\rangle \equiv \frac{1}{N^{N_{\mathrm{imp}}}}\sum_{\boldsymbol{\rho}_1}\sum_{\boldsymbol{\rho}_2}\ldots\sum_{\boldsymbol{\rho}_{N_{\mathrm{imp}}}} Z$, where N is the total number of sites in the system and N_{imp} is the number of defects. Keeping the first two cumulants of the impurity-induced perturbation in the action of the disorder-averaged partition function $\langle Z\rangle$ gives the action

$$\frac{f(s)}{T} = \frac{1}{2T}\sum_{\mathbf{r},\mathbf{r}'} s_{\mathbf{r}}^{\alpha} s_{\mathbf{r}'}^{\alpha}\left(J_{\mathbf{r},\mathbf{r}'} + n_{\mathrm{imp}}k_{\mathbf{r}\mathbf{r}'}\right) - \frac{n_{\mathrm{imp}}}{2T^2}\sum_{\mathbf{r}_1,\mathbf{r}_2,\mathbf{r}_1',\mathbf{r}_2',\alpha,\beta} s_{\mathbf{r}_1}^{\alpha} s_{\mathbf{r}_2}^{\beta}\left(F_{\mathbf{r}_1\mathbf{r}_2\mathbf{r}_1'\mathbf{r}_2'} - n_{\mathrm{imp}}k_{\mathbf{r}_1\mathbf{r}_1'}k_{\mathbf{r}_2\mathbf{r}_2'}\right) s_{\mathbf{r}_1'}^{\alpha} s_{\mathbf{r}_2'}^{\beta}, \tag{2}$$

where we have introduced the ratio $n_{\mathrm{imp}} = N_{\mathrm{imp}}/N$ of the number of defects to the total number of sites in the lattice and the quantities

$$k_{\mathbf{r}\mathbf{r}'} = \sum_{\boldsymbol{\rho}} K_{\mathbf{r}-\boldsymbol{\rho},\mathbf{r}'-\boldsymbol{\rho}}, \tag{3}$$

$$F_{\mathbf{r}_1\mathbf{r}_2\mathbf{r}_1'\mathbf{r}_2'} = \frac{1}{4}\sum_{\boldsymbol{\rho}} K_{\mathbf{r}_1-\boldsymbol{\rho},\mathbf{r}_1'-\boldsymbol{\rho}} K_{\mathbf{r}_2-\boldsymbol{\rho},\mathbf{r}_2'-\boldsymbol{\rho}}. \tag{4}$$

Taking into account higher-order cumulants of the disorder-induced perturbation may change coefficients of order unity in the mean-field phase boundary of the glass phase but will not lead to qualitatively new effects.

Decoupling the variables $s_{\mathbf{r}}^{\alpha} s_{\mathbf{r}'}^{\beta}$ by the Hubbard-Stratonovich field $Q_{\mathbf{r}\mathbf{r}'}^{\alpha\beta}$ gives

$$\begin{aligned}\frac{f(s,Q)}{T} =& \frac{1}{2T} s_{\mathbf{r}}^{\alpha}\tilde{J}_{\mathbf{r},\mathbf{r}'} s_{\mathbf{r}'}^{\alpha} - \frac{n_{\mathrm{imp}}}{2T^2} Q_{\mathbf{r}_1\mathbf{r}_2}^{\alpha\beta}\tilde{F}_{\mathbf{r}_1\mathbf{r}_2\mathbf{r}_1',\mathbf{r}_2'} s_{\mathbf{r}_1'}^{\alpha} s_{\mathbf{r}_2'}^{\beta} \\ &- \frac{n_{\mathrm{imp}}}{2T^2} s_{\mathbf{r}_1}^{\alpha} s_{\mathbf{r}_2}^{\beta}\tilde{F}_{\mathbf{r}_1\mathbf{r}_2\mathbf{r}_1',\mathbf{r}_2'} Q_{\mathbf{r}_1'\mathbf{r}_2'}^{\alpha\beta} + \frac{n_{\mathrm{imp}}}{2T^2} Q_{\mathbf{r}_1\mathbf{r}_2}^{\alpha\beta}\tilde{F}_{\mathbf{r}_1\mathbf{r}_2\mathbf{r}_1',\mathbf{r}_2'} Q_{\mathbf{r}_1'\mathbf{r}_2'}^{\alpha\beta},\end{aligned} \tag{5}$$

where $\tilde{J}_{\mathbf{r},\mathbf{r}'} = J_{\mathbf{r},\mathbf{r}'} + n_{\mathrm{imp}}k_{\mathbf{r}\mathbf{r}'}$ and $\tilde{F}_{\mathbf{r}_1\mathbf{r}_2\mathbf{r}_1'\mathbf{r}_2'} = F_{\mathbf{r}_1\mathbf{r}_2\mathbf{r}_1'\mathbf{r}_2'} - n_{\mathrm{imp}}k_{\mathbf{r}_1\mathbf{r}_1'}k_{\mathbf{r}_2\mathbf{r}_2'}$ and summation over repeated indices is implied. We note that in the limit of low concentrations n_{imp} of the defects, their effect on the quantities $\tilde{J}_{\mathbf{r},\mathbf{r}'} \approx J_{\mathbf{r},\mathbf{r}'}$ and $\tilde{F}_{\mathbf{r}_1\mathbf{r}_2\mathbf{r}_1'\mathbf{r}_2'} \approx F_{\mathbf{r}_1\mathbf{r}_2\mathbf{r}_1'\mathbf{r}_2'}$ in Eq. (5) may be neglected.

Summing over the spin degrees of freedom leads to the free energy as a function of the fields Q given by

$$\begin{aligned}F(Q) =& \frac{n_{\mathrm{imp}}}{2T} Q_{\mathbf{r}_1\mathbf{r}_2}^{\alpha\beta}\tilde{F}_{\mathbf{r}_1\mathbf{r}_2\mathbf{r}_1',\mathbf{r}_2'} Q_{\mathbf{r}_1'\mathbf{r}_2'}^{\alpha\beta} \\ &- \frac{n_{\mathrm{imp}}^2}{8T^3} Q_{\mathbf{r}_1\mathbf{r}_2}^{\alpha\beta}\tilde{F}_{\mathbf{r}_1\mathbf{r}_2\mathbf{r}_1',\mathbf{r}_2'} G_{\mathbf{r}_1'\mathbf{R}_1'} G_{\mathbf{r}_2'\mathbf{R}_2'}\tilde{F}_{\mathbf{R}_1'\mathbf{R}_2'\mathbf{R}_1\mathbf{R}_2} Q_{\mathbf{R}_1\mathbf{R}_2}^{\alpha\beta} \\ &- \frac{n_{\mathrm{imp}}^2}{8T^3} Q_{\mathbf{r}_1\mathbf{r}_2}^{\alpha\beta}\tilde{F}_{\mathbf{r}_1\mathbf{r}_2\mathbf{r}_1',\mathbf{r}_2'} G_{\mathbf{r}_1'\mathbf{R}_1'} G_{\mathbf{r}_2'\mathbf{R}_2'}\tilde{F}_{\mathbf{R}_2'\mathbf{R}_1'\mathbf{R}_1\mathbf{R}_2} Q_{\mathbf{R}_1\mathbf{R}_2}^{\beta\alpha} + \mathcal{O}(Q^3),\end{aligned} \tag{6}$$

where $G_{\mathbf{r}\mathbf{r}'} = \langle s_{\mathbf{r}}^{\alpha} s_{\mathbf{r}'}^{\alpha}\rangle$ is the correlator of spins in the same replica in the pure system. To obtain the phase boundary of the glass phase, we look for the mean-field solution for the fields $Q_{\mathbf{r}_1\mathbf{r}_2}^{\alpha\beta}$.

This solution has the same symmetry $Q^{\alpha\beta}_{\mathbf{r}_1\mathbf{r}_2} = Q^{\alpha\beta}_{\mathbf{r}_2\mathbf{r}_1} = Q^{\beta\alpha}_{\mathbf{r}_1\mathbf{r}_2}$ as the averaged correlators $\langle s^\alpha_{\mathbf{r}_1} s^\beta_{\mathbf{r}_2}\rangle$. Furthermore, although the glass phase breaks replica symmetry[10–12], it is sufficient to look for the replica-symmetric solution to obtain the phase boundary.

Taking into account the symmetry $Q^{\alpha\beta}_{\mathbf{r}_1\mathbf{r}_2} = Q^{\alpha\beta}_{\mathbf{r}_2\mathbf{r}_1} = Q^{\beta\alpha}_{\mathbf{r}_1\mathbf{r}_2}$, the free energy (6) can be rewritten as

$$f(Q) = \frac{n_{\text{imp}}}{2T}\hat{Q}\check{F}\hat{Q} - \frac{n_{\text{imp}}^2}{4T^3}\hat{Q}\check{F}(\hat{G}\otimes\hat{G})\check{F}\hat{Q} + \mathcal{O}(Q^3), \tag{7}$$

where $\hat{Q}$ is our convention for the matrix $Q^{\alpha\beta}_{\mathbf{r}_1\mathbf{r}_2}$ and $\check{F}$ and $\hat{G}\otimes\hat{G}$ are superoperators acting on matrices of the respective dimension; $\hat{Q}\check{F}\hat{Q} \equiv Q^{\alpha\beta}_{\mathbf{r}_1\mathbf{r}_2}\tilde{F}_{\mathbf{r}_1\mathbf{r}_2\mathbf{r}_1',\mathbf{r}_2'}Q^{\alpha\beta}_{\mathbf{r}_1'\mathbf{r}_2'}$ and $\hat{Q}\check{F}(\hat{G}\otimes\hat{G})\check{F}\hat{Q} \equiv Q^{\alpha\beta}_{\mathbf{r}_1\mathbf{r}_2}\tilde{F}_{\mathbf{r}_1\mathbf{r}_2\mathbf{r}_1',\mathbf{r}_2'}G_{\mathbf{r}_1'\mathbf{R}_1'}G_{\mathbf{r}_2'\mathbf{R}_2'}\tilde{F}_{\mathbf{R}_1'\mathbf{R}_2'\mathbf{R}_1\mathbf{R}_2}Q^{\alpha\beta}_{\mathbf{R}_1\mathbf{R}_2}$. The mean-field transition temperature is given by

$$T = \left(\frac{1}{2}n_{\text{imp}}\lambda\right)^{\frac{1}{2}}, \tag{8}$$

where λ is the minimum eigenvalue of the (positive) operator $\check{F}(\hat{G}\otimes\hat{G})$. Because this operator is independent of the the defect concentration in the limit of low defect density n_{imp}, the glass transition temperature vanishes in the limit of vanishing defect concentration.

We emphasise that the result (8) can be applied to models of disorder of various nature, including vacancies in GF systems as well as random-bond disorder, or combinations of different sources of disorder. For weak random-bond disorder on all bonds ($n \sim n_{\text{imp}}$), Eq. (8) predicts a transition temperature proportional to the amplitude of the fluctuation of the exchange coupling, in agreement with the result of Refs. 6 and 7.

III. ON THE POSSIBILITY OF GLASSINESS IN 2D

Some of the GF compounds discussed in our work ($SrCr_8Ga_4O_{19}$, $BaCr_{9-9x}Ga_{3+9x}O_{19}$ and $NiGa_2S_4$) are layered with weak interlayer coupling and, therefore, may be considered as effectively two-dimensional (2D) systems at short distances. In this section, we argue that the 3D nature of those systems, which accounts for the interlayer coupling, is essential to observe the glass transition in them.

There is abundant numerical evidence (see, for example, Refs. 13–18) of the absence of a glass state at a finite temperature $T > 0$ in 2D spin systems with bond disorder. This changes in the presence of field disorder, for a system described by the Hamiltonian

$$\mathcal{H} = \mathcal{H}_0 - \sum_i b_i s_i, \tag{9}$$

where b_i is a random field. In this case, the system has a finite glass order parameter

$$q^{\alpha\beta} = \left\langle s_i^\alpha s_i^\beta \right\rangle = \mathrm{Tr}\left[s_i^\alpha s_i^\beta \exp\left(-\frac{1}{T}\sum_\gamma H_0^\gamma - \frac{\varkappa}{2T^2}\sum_{j,\gamma,\gamma'} s_j^\gamma s_j^{\gamma'} \right)\right] > 0 \tag{10}$$

due to the identity

$$\frac{\partial q^{\alpha\beta}}{\partial \varkappa} = -\frac{1}{T^2} \tag{11}$$

in the limit of weak disorder strength $\varkappa = \langle b_j^2 \rangle_{\mathrm{dis}}$. The presence of a finite glass order parameter in a system with random-field disorder is similar, in some sense, to the existence of a finite magnetisation in a magnet in a magnetic field. Because the order parameter $q^{\alpha\beta}$ is nonzero at all temperatures, there is no phase transition between the glass ($q^{\alpha\beta} \neq 0$) and non-glass ($q^{\alpha\beta} = 0$) phases.

Both in the case of bond- and random-field disorder, the glass transition is absent; the system either has the glass order parameter at all temperatures or never has it. Because the glass transition is observed in all geometrically frustrated compounds discussed in the main text of this paper, the three-dimensional nature of the discussed layered compounds ($SrCr_8Ga_4O_{19}$, $BaCr_{9-9x}Ga_{3+9x}O_{19}$ and $NiGa_2S_4$) is, therefore, important for the existence of the transition.

IV. CROSSOVER IN THE MAGNETIC PERMEABILITY IN THE COULOMB PHASE

In this section, we describe the mechanism of a crossover in the behaviour of a GF medium that drives the glass transition in a spin ice or a system of classical vector spins. Such a crossover is a property of the clean system and, albeit insufficient to cause a transition on its own, determines the conditions favourable for the onset of the glass phase.

We assume that the medium is described by the Coulomb phase[19–23]. The role of the variables in this description is played by a coarse-grained classical field $\mathbf{B}$ associated with the polarisations of the spins. In the absence of impurities and interactions other than the exchange interactions between the spins, the classical ground states of the system (states that minimise the total exchange energy) are hugely degenerate, corresponding to all configurations of the field B that satisfy the condition $\mathrm{div}\mathbf{B} = 0$.

Flipping a spin creates a pair of monopoles with opposite charges that can be further separated by local rotations of the spins. Creating an isolated monopole requires a large energy of the order of the AF coupling and will not be central to our arguments here. Defects with spins that mismatch

the spins of the parent compound also correspond to monopole charges Q_i at the locations of the defects.

Interactions other than the antiferromagnetic exchange interactions between the spins may lift the degeneracy of the abovementioned classical ground states. Here, we assume that such interactions are present and, in the limit of small B, lead to the temperature-independent contribution $\frac{1}{8\pi}\int \mathbf{B}^2 d\mathbf{r}$ (measured in appropriate units) to the free energy of the system. It may come, for example, from the magnetic dipole-dipole interactions between the spins or from interactions beyond nearest neighbours[24]. Another possible mechanism for generating this energy is quantum tunnelling[25–27] between classical ground states of the regions with $\mathrm{div}\mathbf{B} = 0$. While it does not require the existence of an energy scale other than the exchange energy, the amplitude of the tunnelling is in general exponentially suppressed by the number of spins that need to be rotated in order to arrive from one classical ground state to another [17-19]. The exact nature of the energy of the field $\mathbf{B}$ is not important for our consideration and cannot be uniquely inferred from the available data, which is why we leave the investigation of the origin of this energy for future studies.

The free energy of the systems also includes the "entropic" contribution[22] $\frac{1}{8\pi}\frac{T}{\tilde{T}}\int \mathbf{B}^2 d\mathbf{r}$ linear in the temperature T, scaled by a constant prefactor $1/\tilde{T}$. This contribution accounts for the decrease of the entropy of the systems with increasing the parameter $\mathbf{B}$.

Combining all the discussed contributions gives the free energy of low-energy excitations in a GF material in the form

$$F(\phi,\mathbf{B}) = \frac{1}{8\pi}\frac{T+\tilde{T}}{\tilde{T}}\int \mathbf{B}^2 d\mathbf{r} - \frac{1}{4\pi}\int \phi\, \mathrm{div}\mathbf{B}\, d\mathbf{r} + \sum_i \left[\phi(\mathbf{r}_i) + E_Q\right] Q_i - \int \mathbf{h}\cdot\mathbf{B} d\mathbf{r} - \frac{1}{8\pi}\int h_0 \mathbf{B}^2\, d\mathbf{r} \quad (12)$$

where Q_i and $\mathbf{r}_i$ are the charges and the locations of the monopoles introduced by the defects; E_Q is the energy of the defect, which may depend on the state of the defect spin; $\mathbf{h}(\mathbf{r})$ and $h_0(\mathbf{r})$ are weak quenched random fields that account for disorder other than monopole charges created by magnetic vacancies, e.g. random strain. In Eq. (S1), we have introduced the field ϕ, minimisation with respect to which enforces the Gauss theorem in the form $\mathrm{div}\mathbf{B} = 4\pi\sum_i Q_i\delta(\mathbf{r}-\mathbf{r}_i)$. For states that minimise the free energy (12), the scalar field ϕ plays the role of the potential of the Coulomb field, $\boldsymbol{\nabla}\phi = -\frac{T+\tilde{T}}{\tilde{T}}\mathbf{B} + 4\pi\mathbf{h} + h_0\mathbf{B}$, where the random fields $4\pi\mathbf{h}$ and $h_0\mathbf{B}$ come from the fluctuations of the effective magnetisation and magnetic permeability. The free energy (S1) describes a system of randomly located magnetic charges (defects) in a disordered medium with the average magnetic permeability determined by the properties of the disorder-free system and

given by Eq. (1) in the main text.

We emphasise that the permeability $\mu(T)$ given by Eq. (1) characterises the interaction between excitations in the system and is in general unrelated to the magnetic susceptibility $\chi(T)$, which characterises response to the external magnetic field. The energy $E \approx E_0 + \frac{1}{\mu(T)} \sum_{i,j<i} \frac{Q_i Q_j}{|\mathbf{r}_i - \mathbf{r}_j|}$ of a system of monopoles in the material consists of a temperature-independent contribution E_0 determined by the exchange coupling between spins and the interaction contribution $\propto [\mu(T)]^{-1}$. The temperature dependence of the permeability $\mu(T)$ can thus be independently verified via spin resonance spectroscopy of multi-charge, e.g, dipole, excitations in the system.

The glass state in the system described by the free energy (S1) requires sufficiently strong disorder represented by randomly located impurities, which create monopole charges Q_i, or other sources of randomness represented by the quenched parameters $\mathbf{h}$ and h_0 in Eq. (1). We emphasise that the hidden energy scale T^*, the glass transition temperature at zero impurity concentration, cannot be explained solely by the residual disorder distinct from impurities because the glass transition temperature grows with decreasing disorder. This trend, the exact microscopic mechanism of which we leave for future studies, suggests also a significant role of the disorder-free medium for the "hidden" energy scale T^* discussed in this paper.

---

[1] P. Schiffer and I. Daruka, "Two-population model for anomalous low-temperature magnetism in geometrically frustrated magnets," Phys. Rev. B **56**, 13712–13715 (1997).

[2] Arnab Sen, Kedar Damle, and Roderich Moessner, "Fractional Spin Textures in the Frustrated Magnet $SrCr_{9p}Ga_{12-9p}O_{19}$," Phys. Rev. Lett. **106**, 127203 (2011).

[3] A. D. LaForge, S. H. Pulido, R. J. Cava, B. C. Chan, and A. P. Ramirez, "Quasispin glass in a geometrically frustrated magnet," Phys. Rev. Lett. **110**, 017203 (2013).

[4] Arnab Sen and R. Moessner, "Topological spin glass in diluted spin ice," Phys. Rev. Lett. **114**, 247207 (2015).

[5] W. Ratcliff, S.-H. Lee, C. Broholm, S.-W. Cheong, and Q. Huang, "Freezing of spin correlated nanoclusters in a geometrically frustrated magnet," Phys. Rev. B **65**, 220406 (2002).

[6] A. Andreanov, J. T. Chalker, T. E. Saunders, and D. Sherrington, "Spin-glass transition in geometrically frustrated antiferromagnets with weak disorder," Phys. Rev. B **81**, 014406 (2010).

[7] T. E. Saunders and J. T. Chalker, "Spin freezing in geometrically frustrated antiferromagnets with weak disorder," Phys. Rev. Lett. **98**, 157201 (2007).

[8] M. Sato and J.E. Greedan, "Pyrochlore solid solutions $(La_xY_{1-x})2Mo2O7$, $x = 0.0$ to 0.5 Spin-glass-like

behavior," Journal of Solid State Chemistry **67**, 248–253 (1987).

[9] H. Martinho, N. O. Moreno, J. A. Sanjurjo, C. Rettori, A. J. García-Adeva, D. L. Huber, S. B. Oseroff, W. Ratcliff, S.-W. Cheong, P. G. Pagliuso, J. L. Sarrao, and G. B. Martins, "Magnetic properties of the frustrated antiferromagnetic spinel $ZnCr_2O_4$ and the spin-glass $Zn_{1-x}Cd_xCr_2O_4$ ($x = 0.05, 0.10$)," Phys. Rev. B **64**, 024408 (2001).

[10] Marc Mézard, Giorgio Parisi, and Miguel Angel Virasoro, *Spin glass theory and beyond: An Introduction to the Replica Method and Its Applications*, Vol. 9 (World Scientific Publishing Company, 1987).

[11] Viktor S Dotsenko, "Physics of the spin-glass state," **36**, 455–485 (1993).

[12] K. Binder and A. P. Young, "Spin glasses: Experimental facts, theoretical concepts, and open questions," Rev. Mod. Phys. **58**, 801–976 (1986).

[13] W. L. McMillan, "Domain-wall renormalization-group study of the three-dimensional random Ising model," Phys. Rev. B **30**, 476–477 (1984).

[14] A. K. Hartmann and A. P. Young, "Lower critical dimension of Ising spin glasses," Phys. Rev. B **64**, 180404 (2001).

[15] A. C. Carter, A. J. Bray, and M. A. Moore, "Aspect-Ratio Scaling and the Stiffness Exponent θ for Ising Spin Glasses," Phys. Rev. Lett. **88**, 077201 (2002).

[16] C. Amoruso, E. Marinari, O. C. Martin, and A. Pagnani, "Scalings of domain wall energies in two dimensional ising spin glasses," Phys. Rev. Lett. **91**, 087201 (2003).

[17] L. A. Fernandez, E. Marinari, V. Martin-Mayor, G. Parisi, and J. J. Ruiz-Lorenzo, "Universal critical behavior of the two-dimensional Ising spin glass," Phys. Rev. B **94**, 024402 (2016).

[18] Heiko Rieger, Ludger Santen, Ulrich Blasum, Martin Diehl, Michael Jünger, and Giovanni Rinaldi, "The critical exponents of the two-dimensional Ising spin glass revisited: exact ground-state calculations and Monte Carlo simulations," Journal of Physics A: Mathematical and General **29**, 3939 (1996).

[19] S. V. Isakov, K. Gregor, R. Moessner, and S. L. Sondhi, "Dipolar spin correlations in classical pyrochlore magnets," Phys. Rev. Lett. **93**, 167204 (2004).

[20] C. L. Henley, "Power-law spin correlations in pyrochlore antiferromagnets," Phys. Rev. B **71**, 014424 (2005).

[21] Michael Hermele, Matthew P. A. Fisher, and Leon Balents, "Pyrochlore photons: The $u(1)$ spin liquid in a $s = \frac{1}{2}$ three-dimensional frustrated magnet," Phys. Rev. B **69**, 064404 (2004).

[22] Christopher L. Henley, "The "Coulomb Phase" in Frustrated Systems," Annual Review of Condensed Matter Physics **1**, 179–210 (2010), https://doi.org/10.1146/annurev-conmatphys-070909-104138.

[23] Lucile Savary and Leon Balents, "Quantum spin liquids: a review," **80**, 016502 (2016).

[24] Villain, J. and Loveluck, J.M., "Néel temperature of a low-dimensional antiferromagnet in a magnetic field," J. Physique Lett. **38**, 77–80 (1977).

[25] Jan von Delft and Christopher L. Henley, "Spin tunneling in the kagomé antiferromagnet," Phys. Rev. B **48**, 965–984 (1993).

[26] K. Iida, S.-H. Lee, and S.-W. Cheong, "Coexisting order and disorder hidden in a quasi-two-dimensional

frustrated magnet," Phys. Rev. Lett. **108**, 217207 (2012).

[27] Junjie Yang, Anjana Samarakoon, Sachith Dissanayake, Hiroaki Ueda, Israel Klich, Kazuki Iida, Daniel Pajerowski, Nicholas P. Butch, Q. Huang, John R. D. Copley, and Seung-Hun Lee, "Spin jam induced by quantum fluctuations in a frustrated magnet," Proceedings of the National Academy of Sciences **112**, 11519–11523 (2015), https://www.pnas.org/content/112/37/11519.full.pdf.
